# The Coli Surface Antigen CS3 of Enterotoxigenic *Escherichia coli* Is Differentially Regulated by H-NS, CRP, and CpxRA Global Regulators

**DOI:** 10.3389/fmicb.2019.01685

**Published:** 2019-07-25

**Authors:** Miguel A. Ares, Judith Abundes-Gallegos, Diana Rodríguez-Valverde, Leonardo G. Panunzi, César Jiménez-Galicia, Ma. Dolores Jarillo-Quijada, María Lilia Cedillo, Marìa D. Alcántar-Curiel, Javier Torres, Jorge A. Girón, Miguel A. De la Cruz

**Affiliations:** ^1^Unidad de Investigación Médica en Enfermedades Infecciosas y Parasitarias, Hospital de Pediatría, Centro Médico Nacional Siglo XXI, Instituto Mexicano del Seguro Social, Mexico City, Mexico; ^2^Institut Pasteur, Biodiversity and Epidemiology of Bacterial Pathogens, Paris, France; ^3^Unidad Médica de Alta Especialidad, Laboratorio Clínico, Hospital de Pediatría, Centro Médico Nacional Siglo XXI, Instituto Mexicano del Seguro Social, Mexico City, Mexico; ^4^Unidad de Investigacioìn en Medicina Experimental, Facultad de Medicina, Universidad Nacional Autónoma de México, Mexico City, Mexico; ^5^Centro de Detección Biomolecular, Benemérita Universidad Autónoma de Puebla, Puebla, Mexico

**Keywords:** CS3, H-NS, CRP, CpxRA, enterotoxigenic *E. coli*

## Abstract

Enterotoxigenic *Escherichia coli* produces a myriad of adhesive structures collectively named colonization factors (CFs). CS3 is a CF, which is assembled into fine wiry fibrillae encoded by the *cstA-H* gene cluster. In this work we evaluated the influence of environmental cues such as temperature, osmolarity, pH, and carbon source on the expression of CS3 genes. The transcription of *cstH* major pilin gene was stimulated by growth of the bacteria in colonization factor broth at 37°C; the presence of glycerol enhanced *cstH* transcription, while glucose at high concentration, high osmolarity, and the depletion of divalent cations such as calcium and magnesium repressed *cstH* expression. In addition, we studied the role of H-NS, CpxRA, and CRP global regulators in CS3 gene expression. H-NS and CpxRA acted as repressors and CRP as an activator of *cstH* expression. Under high osmolarity, H-NS, and CpxRA were required for *cstH* repression. CS3 was required for both, bacterial adherence to epithelial cells and biofilm formation. Our data strengthens the existence of a multi-factorial regulatory network that controls transcription of CS3 genes in which global regulators, under the influence of environmental signals, control the production of this important intestinal colonization factor.

## Introduction

Enterotoxigenic *Escherichia coli* (ETEC) is responsible for high rates of morbidity and mortality in children living in countries with poor sanitation and is the main cause of travelers’ diarrhea (Khalil et al., 2018; Mirhoseini et al., 2018). The most important virulence factors of ETEC are the heat-labile (LT) and heat-stable (ST) enterotoxins, which are responsible for the secretory watery diarrhea that characterizes human ETEC infection (Fleckenstein et al., 2010). Colonization of the small intestine is achieved by adhesive pili collectively named colonization factors (CFs), which also include the coli surface antigens (CS1–CS6) (Qadri et al., 2005; Fleckenstein et al., 2010; Madhavan and Sakellaris, 2015). Twenty-three different CFs have been described so far and their distribution in ETEC strains varies according to geographic regions (Madhavan and Sakellaris, 2015). One of the most common CFs is CS3, which can be co-produced with CS1 or CS2 by some ETEC strains (Begum et al., 2014; Vidal et al., 2019). Human volunteers fed with the CS3-producing strain E9034A developed diarrhea and showed significant rises in serum IgG antibody against CS3, suggesting that this adhesin is produced *in vivo*, plays a role in pathogenesis, and triggers an antibody immune response (Levine et al., 1984; Knutton et al., 1985).

In contrast to most CFs, which are long rod-like pili, the CS3 surface antigen assembles into fine wiry fibrillae (2–3 nm wide). The production of CS3 is encoded by the *cstA-H* gene cluster (Manning et al., 1985; Boylan et al., 1987), where the *cstA* gene codes for a putative chaperone protein which shares homology to FimC and PapD proteins that participate in the assembly of the type I and P pili, respectively (Yakhchali and Manning, 1997). The *cstB* gene codes for an usher protein and the four *cstC-F* genes are contained within the same *cstB* ORF each having different translational start points and only one stop codon (Jalajakumari et al., 1989). Finally, both *cstG* and *cstH* genes code for pilin subunits (Hall et al., 1989; Jalajakumari et al., 1989), of which CstH is the major pilin subunit.

Host and environmental stimuli such as temperature, osmolarity, pH, and carbon source are sensed by ETEC to regulate the expression of virulence factors such as toxins and pili (Kansal et al., 2013; Munson, 2013; Haycocks et al., 2015; De La Cruz et al., 2017). However, only in a few cases the influence of environmental and nutritional factors on the expression and production of some of the ETEC’s CFs has been reported. ETEC possesses a myriad of transcription regulators controlling its virulence. Namely, H-NS (histone-like nucleoid structuring), CpxRA (conjugation pilus expression), and CRP (cAMP receptor protein) are global regulators that control the expression of the main virulence factors in ETEC, such as enterotoxins and CFs (Yang et al., 2005; Mellies and Barron, 2006; Bodero and Munson, 2009; Haycocks et al., 2015; De La Cruz et al., 2017). H-NS is a pleiotropic regulator, which binds AT-rich DNA regions blocking the interaction of the RNA polymerase, silencing transcription of housekeeping, and virulence genes (Dorman, 2007). CRP is a global regulatory protein involved in catabolic repression in many enterobacteria and its activity depends of the binding of cAMP to target protein (Kolb et al., 1993). The CpxRA two-component system senses a variety of stimuli (e.g., pH changes, overexpression of envelope proteins, and alterations in the membrane) within the bacterial cell envelope, controlling virulence in enterobacterial pathogens (Vogt and Raivio, 2012).

In this work we sought to investigate the environmental cues and transcriptional factors involved in the regulation of CS3 using the prototypic ETEC strain E9034A. In particular, we inquired about the role of global regulators such as H-NS, CpxRA, and CRP, in the transcriptional control of CS3. In addition, the function of CS3 in both bacterial adherence to epithelial cells and biofilm formation was analyzed using an isogenic *cstH* mutant. The data generated in this work will help to understand the regulation of this important intestinal CF and to suggest a model of the regulatory network that controls ETEC virulence factors.

## Materials and Methods

### Bacterial Strains and Growth Conditions

Enterotoxigenic *Escherichia coli* strains used and bacterial constructs generated in this study are listed in Table 1. E9034A [CS3^+^, Longus (CS21)^+^, LT^+^, ST^+^] was used as the prototypic strain and for construction of isogenic mutants (Levine et al., 1984; Giron et al., 1994). For routine work, the strains were grown overnight in Luria-Bertani broth (LB) with shaking at 37°C. To determine expression of *cst* genes, several liquid bacteriological media such as pleuropneumoniae-like organisms (PPLO), LB, trypticase soy broth (TSB), *Brucella* broth (BB), colonization factor broth (CFA), Dulbecco’s modified Eagle’s medium (DMEM) with low (1.0 g/l) or high glucose (4.5 g/l), and brain heart infusion (BHI) were used. Environmental conditions were tested to analyze the transcription of *cstH* gene in CFA broth at 37°C with shaking and samples were collected 6 h post-inoculation for RNA extraction. CFA broth was prepared as previously described [1% Casamino Acids and 0.15% yeast extract plus 0.005% MgSO_4_ and 0.0005% MnCl_2_ (Evans et al., 1977)]. In addition, CFA broth supplemented with 0.3M NaCl, 1.0 mM EDTA, 5.0 mM CaCl_2_, 5.0 mM MgCl_2_, 0.05% glucose or 0.05% glycerol was prepared.

**TABLE 1:** List of bacterial strains and plasmids used.

**Strain or plasmid**	**Genotype or description**	**References or source**
**ETEC strains**		
ETEC WT	ETEC strain E9034A, LT^+^, ST^+^, CS21^+^, CS3^+^	Levine, 1987
ETEC Δ*hns*	ETEC Δ*hns*::Km^R^	De La Cruz et al., 2017
ETEC Δ*cpxRA*	ETEC Δ*cpxRA*::FRT	De La Cruz et al., 2017
ETEC Δ*crp*	ETEC Δ*crp*::Km^R^	De La Cruz et al., 2017
ETEC ΔcstH	ETEC Δ*cstH*::Km^R^	This study
**Plasmids**		
pMPM-T3	p15A derivative low-copy-number cloning vector, *lac* promoter; Tc^R^	Mayer, 1995
pT3-CstH	pMPM-T3 derivative expressing CstH from the *lac* promoter	This study
pT3-H-NS	pMPM-T3 derivative expressing H-NS from the lac promoter	De La Cruz et al., 2017
pSUcrp	pSU19 carrying a *crp* gene	Guo et al., 2015
pBAD-CpxA	pBAD/*Myc*-His A derivative expressing CpxA-*Myc*-His_6_ from the pBAD promoter	De La Cruz et al., 2016
pMPM-K6	p15A derivative cloning vector, pBAD (*ara*) promoter; Km^R^	Mayer, 1995
pK6-CpxR	pMPM-K6 derivative expressing His_6_-CpxR from the pBAD promoter	This study
pKD46	pINT-ts derivative containing the λ Red recombinase system under an arabinose-inducible promoter, Ap^R^	Datsenko and Wanner, 2000
pKD4	pANTsγ derivative template plasmid containing the kanamycin cassette for λ Red recombination, Ap^R^	Datsenko and Wanner, 2000

### Construction of Isogenic Mutants and Plasmids

Enterotoxigenic *Escherichia coli* Δ*cstH* mutant was generated by the lambda Red recombinase mutagenesis system as previously described (Datsenko and Wanner, 2000), using gene-specific primer pairs to amplify the kanamycin resistance gene from plasmid pKD4 (Table 1). For cloning of *cstH* and complementation of theΔ*cstH* mutant, specific primers (Table 2) containing the *Hind*III (5′)/*Bam*HI (3′) restriction sites were used to obtain a PCR product, which was digested with *Hind*III and *Bam*HI restriction enzymes and then ligated into pMPM-T3 previously digested with the same restriction enzymes. For *cpxR*, primers (Table 2) containing the *Nco*I (5′)/*Hind*III (3′) restriction sites were used to generate a PCR product, which was digested with *Nco*I and *Hind*III and then ligated into pMPM-K6 (Mayer, 1995) previously digested with the same restriction enzymes, generating pK6-CpxR. The mutations and clones were confirmed by PCR and nucleotide sequencing.

**TABLE 2:** Primers used in this study.

**Primer**	**Sequence (5′–3′)**
**For gene deletion**	
cstH-H1P1	AGT GAA TGT ATG AGG GAT TCG ATG TTA AAA ATA AAA TAC TTA TTA TGT AGG CTG GAG CTG CTT CG
cstH-H2P2	AAG GCT ACG TCT ATT ATA TAA TTA TTT AAT TGT CGA AGT AAT TGT CAT ATG AAT ATC CTC CTT AG
**For mutant characterization**	
cstH-F	GGT CTT AAC GTA ACC AGT AAT G
cstH-R	CAT AAA GAT AGC AAC GTA GCC
**For gene cloning**	
cstH-HindIII-F	GGG AAG CTT GCT ACA TGC ACA CAG GAG TAG CAG TAC
cstH-BamHI-R	CCC GGA TCC GGA GCA GAA TTA CAA GCT TGA C
cpxR-NcoI-6His-F	AAA CCA TGG ATC ACC ACC ACC ACC ACC ACA AAA TCC TGT TAG TTG ATG ATG
cpxR-HindIII-R	CGG TTC GAA ACC GCT TCT ACG CGC GCC AAT TC
**For qPCR**	
cstA-F	AGG GCC GTA TGA GCA AAA AC
cstA-R	TCA CTG CTC TCA CCT AGA TCA C
cstB-F	CGG TCT TGC GCC AAT TAA AC
cstB-R	AGC GTG GTT GAA TGT TAG CG
cstG-F	GCG TTT CTG ACA ACT CTG CA
cstG-R	TCC CCA CAC CGA CAT CAA AG
cstH-F	AGC TTT GCC ACC ACC ATT TC
cstH-R	TGG CAA CTG ACT CCC ATT TG
rrsH-F	TGC ATC TGA TAC TGG CAA GC
rrsH-R	TAC GCA TTT CAC CGC TAC AC

### Quantitative RT-PCR

Total RNA was extracted from bacteria grown under different culture conditions using the hot phenol method (Jahn et al., 2008). DNA was removed with TURBO DNA-free (Ambion, Inc.) and the quality of RNA was assessed using a NanoDrop (ND-1000; Thermo Scientific) and an Agilent 2100 bioanalyzer with a Picochip (Agilent Technologies). The absence of contaminating DNA was controlled by the lack of amplification products after 35 qPCR cycles using RNA as template. Control reactions with no template (water) and without reverse transcriptase were rn in all experiments. cDNA synthesis and qPCR was performed as previously described (Ares et al., 2016; De La Cruz et al., 2016). Specific primers were designed with the Primer3Plus software and they are listed in Table 2. 16S rRNA (*rrsH*) was used as a reference gene for normalization and the relative gene expression was calculated using the 2^– ΔΔCt^ method (Livak and Schmittgen, 2001).

### Bacterial Infection and Adherence Assays

Monolayers of HeLa (ATCC CCL-2) human cervix epithelial and Caco-2 (ATCC HTB-37) human colonic epithelial cell lines were used in adherence assays as previously described (De La Cruz et al., 2016). For infection, these cells were cultivated in DMEM high glucose (4.5 g/l) (Invitrogen) at 37°C under a 5% CO_2_ atmosphere in polystyrene 24-well plates (CellStar). Next day, the bacteria (previously grown in LB overnight), were subcultured in CFA broth at early stationary phase (OD_600 nm_ = 1.2) at 37°C. The cell monolayers (7 × 10^5^) were infected at a multiplicity of infection (MOI) of 100 for 2 h, washed thrice with PBS to remove unbound bacteria, and subsequently treated with 1 mL of 0.1% Triton X-100 for 15 min. Following lysis, colony-forming-units (CFUs) were obtained and quantified by plating out 10-fold dilutions of the bacterial suspensions. The experiments were performed in triplicate on three different days, and the mean results were expressed as adhering CFUs/ml.

### Biofilm Assay

Overnight bacterial cultures were diluted 1:100 with CFA broth and 200 μL aliquots were transferred to 96-well plates (Nunc, Sigma-Aldrich). After incubation for 24 h at 30°C the medium was discarded and the wells were rinsed thrice with PBS. The biofilms were stained with 1% Crystal Violet (Merck). After washing, the adsorbed dye was recovered with ethanol and the color read at an optical density of 595 nm with a spectrophotometer (Multiskan Ascent, Thermo Scientific). All the samples were tested in quintupled and the experiments were repeated three times on different days. The data represent the average of the mean of three experiments.

### Statistical Analysis

For statistical differences, one-way ANOVA followed by the Tukey’s comparison test was performed using Prism5.0 (GraphPad Software, Inc., San Diego, CA, United States). *p* ≤ 0.05 was considered statistically significant.

## Results

### Genetic Organization of CS3

**FIGURE 1:**
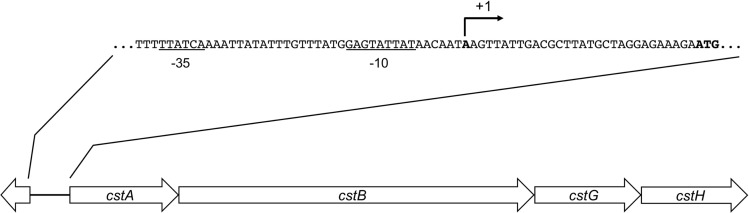
Genetic organization of CS3. Schematic representation of the CS3-encoding region. Arrows indicate coding sequences and lines represent intergenic regions. By using BPROM, putatives –10 and –35 boxes (underlined nucleotides) and a putative transcription start site (broken arrow) were identified.

Similar to other CFs, the assembly of CS3 is directed by genes encoded on a large virulence plasmid of ETEC (Madhavan and Sakellaris, 2015). This locus is a 4.7-kbp region encoding four genes: *cstA*, *cstB*, *cstG*, and *cstH* (Figure 1). Using bioinformatics analysis, we found a putative promoter region located upstream of *cstA* gene, which appears to be transcriptionally organized in a polycistronic operon (Figure 1). The CS3-encoding region found in the sequenced ETEC strain 1392/75 showed that it has a GC content of 35.6%, which is significantly different to the average 47.7 and 50.7% of the p1018 plasmid and the chromosome, respectively, suggesting that CS3 genes were acquired by horizontal transfer.

### CS3 Is Expressed in CFA Broth at Early Stationary Phase

**FIGURE 2:**
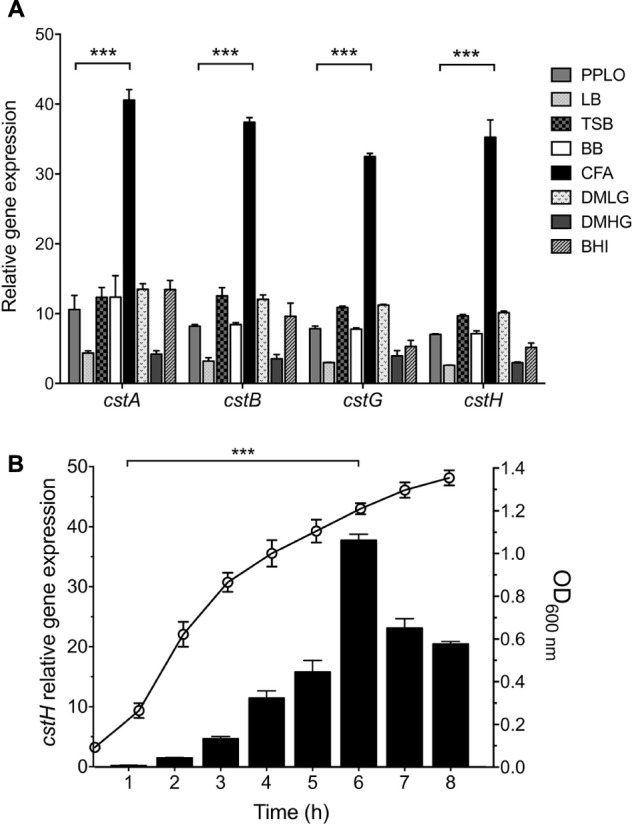
CS3 is expressed in CFA broth. **(A)** Transcription (RT-qPCR) of the CS3 genes at 6 h in several broth: PPLO, LB, TSB, BB, DMEM with low (1.0 g/L) or high glucose (4.5 g/L), and BHI. **(B)** Expression of *cstH* during growth phases as determined by RT-qPCR. This graph represents the mean of three separate experiments performed with triplicate samples with standard deviations. Statistically significant with respect to PPLO broth **(A)** or the bacteria grown after 1 h post-inoculation in CFA broth **(B)**. ^∗∗∗^*p* < 0.001.

Colonization factor broth agar was reported to induce the production of CFs of ETEC (Evans et al., 1977; Giron et al., 1995). We began this study by determining transcription of *cstA*, *cstB*, *cstG*, and *cstH* by RT-qPCR, at early stationary phase (OD_600 nm_ = 1.2) of E9034A grown at 37°C in different culture media. In agreement with the literature, our data showed that the expression of CS3 *cstABGH* genes was significantly induced in CFA broth (Figure 2A) as compared to growth in other rich media such as LB, PPLO, or DMEM defined medium. The level of expression of the different *cst* genes in the different culture media, suggest that they are genetically organized in an operon (Figures 1, 2A). Next, we analyzed transcription of the *cstH* upon growth of the bacteria in CFA broth during 8 h at 37°C. The expression of *cstH* reached its highest level after 6 h of growth, which corresponds to early stationary growth phase (OD_600 nm_ = 1.2) (Figure 2B).

### Effect of Temperature and Nutritional Components on CS3 Expression

**FIGURE 3:**
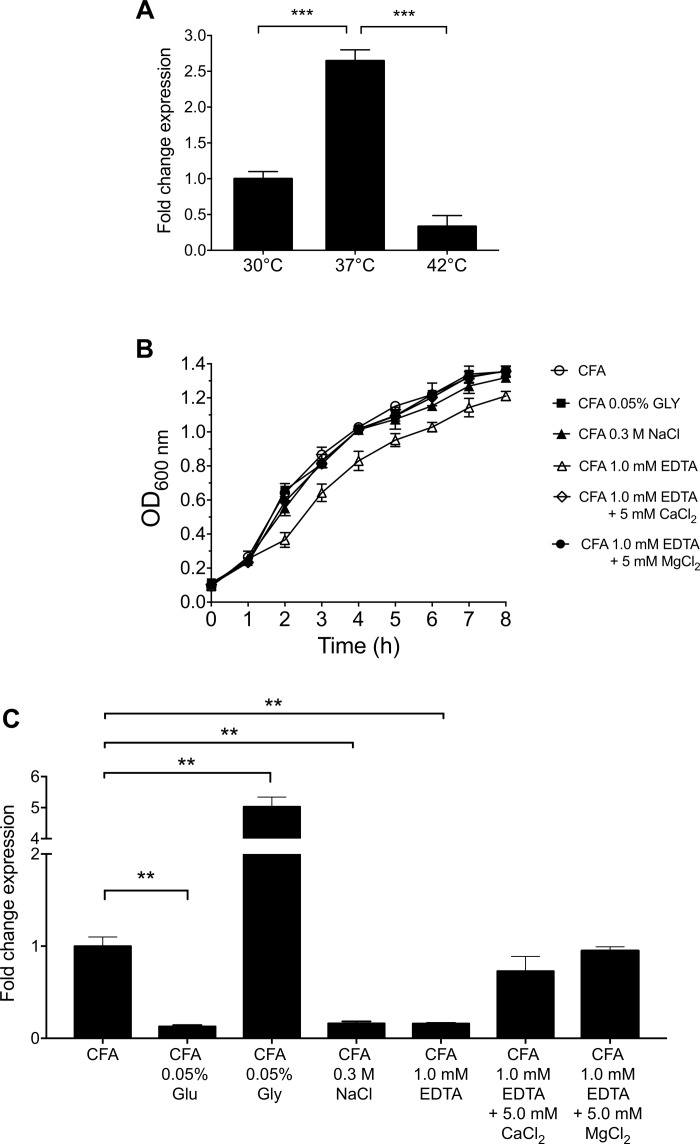
Environmental cues that affect the expression of CS3. **(A)** Expression of CS3 was determined by RT-qPCR when ETEC was grown at different temperatures. **(B)** Growth kinetics of wild-type ETEC under different environmental conditions in CFA broth at 37°C for 8 h, supplemented with glucose (Glu, 0.05%), glycerol (Gly, 0.05%), ethylenediaminetetraacetic acid (EDTA, 1.0 mM, supplemented or not with 5.0 mM CaCl_2_/MgCl_2_), and sodium chloride (NaCl, 0.3M). **(C)** Transcription of *cstH* gene in CFA broth at early stationary phase (OD_600 nm_ = 1.2) at 37°C. Results shown represent mean and standard deviations of three independent experiments performed. Statistically significant with respect to the bacteria grown at 37°C **(A)** or CFA broth not supplemented **(B)**. ^∗∗^*p* < 0.01; ^∗∗∗^*p* < 0.001.

The expression of CS3 was analyzed at different temperatures. The highest level of expression of *cstH* was seen at 37°C as compared to growth at 30 and 42°C in CFA broth at early stationary phase (OD_600 nm_ = 1.2) (Figure 3A), which is a common feature for virulence determinants in human enteric pathogens.

Because the expression of virulence factors is influenced by the carbon source, divalent cations and osmolarity (Bilecen and Yildiz, 2009; Rothenbacher and Zhu, 2014; De La Cruz et al., 2017), we analyzed the effect of the addition of glucose, glycerol, EDTA, and sodium chloride to CFA broth on the expression of *cstH* at early stationary phase (OD_600 nm_ = 1.2) at 37°C. The depletion of divalent cations by addition of EDTA affected ETEC growth and reduced *cstH* transcription and this defect was reversed by the addition of calcium and magnesium cations to the CFA broth (Figures 3B,C). Glucose and glycerol significantly repressed and activated the expression of *cstH*, respectively (Figure 3C). Lastly, high osmolarity (0.3M NaCl) also affected negatively the transcription of the *cstH* gene (Figure 3C). Our data show that environmental conditions such as high osmolarity, high glucose and the depletion of divalent cations repress CS3 gene transcription.

### H-NS, CRP, and CpxRA Control the Expression of CS3

**FIGURE 4:**
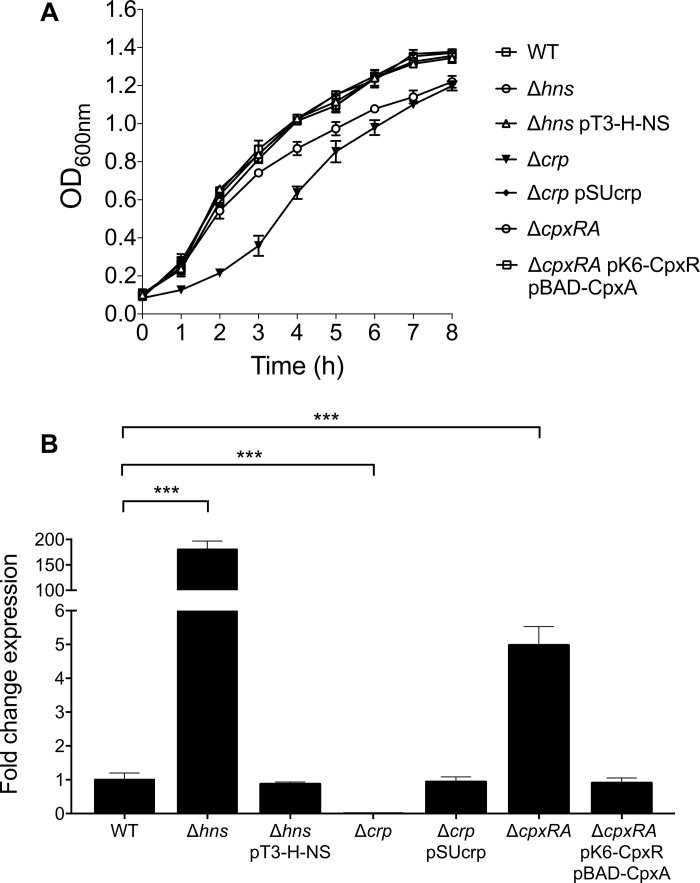
Role of H-NS, CRP, and CpxRA on CS3 expression. **(A)** Growth kinetics of wild-type ETEC, Δ*hns*, Δ*hns* pT3-H-NS, Δ*crp*, Δ*crp* pSUcrp, Δ*cpxRA* and Δ*cpxRA* pK6-CpxR pBAD-CpxA, in CFA broth at 37°C. Bacterial cultures were grown for 8 h in CFA broth. **(B)** Determination of transcriptional expression (RT-qPCR) of the *cstH* gene in CFA broth at early stationary phase (OD_600 nm_ = 1.2) at 37°C in several backgrounds: wild-type (E9034A), Δ*hns*, Δ*hns* pT3-H-NS, Δ*crp*, Δ*crp* pSUcrp, Δ*cpxRA*, and Δ*cpxRA* pK6-CpxR pBAD-CpxA. Results shown represent mean and standard deviations of three independent experiments performed. Statistically significant with respect to the wild-type strain. ^∗∗∗^*p* < 0.001.

The expression of virulence genes in ETEC is regulated by complex networks involving several global transcriptional regulators such as H-NS, CpxRA, and CRP (Kansal et al., 2013; Haycocks et al., 2015; De La Cruz et al., 2017). For instance, we recently reported that the expression of Longus type IV pilus in ETEC was regulated by such global regulators (De La Cruz et al., 2017). We sought to investigate if these global regulators were implicated in the regulatory network controlling CS3 gene expression. To determine the expression of *cstH*, E9034A isogenic *hns*, *crp*, and *cpxRA* mutants were analyzed by RT-qPCR in CFA broth at early stationary phase (OD_600 nm_ = 1.2) at 37°C. Of note, growth of both *hns* and *crp* mutants was affected in CFA broth and the complemented mutants showed similar growth to the wild-type strain (Figure 4A). The transcription of *cstH* was drastically increased and diminished in the *hns* (180-fold) and *crp* (100-fold) mutants, respectively, as compared to the wild-type background (Figure 4B). Interestingly, in the absence of CpxRA the expression of *cstH* was up-regulated (fivefold) as compared to the wild-type strain. These effects were counteracted when the mutant strains were trans-complemented with *hns*, *crp*, and *cpxRA* genes carried on plasmids (Figure 4B). These data are compelling evidence that H-NS, CRP, and CpxRA regulate transcription of CS3 genes.

### Effect of H-NS and CpxRA on the Osmo- and Thermo-Regulation of CS3

**FIGURE 5:**
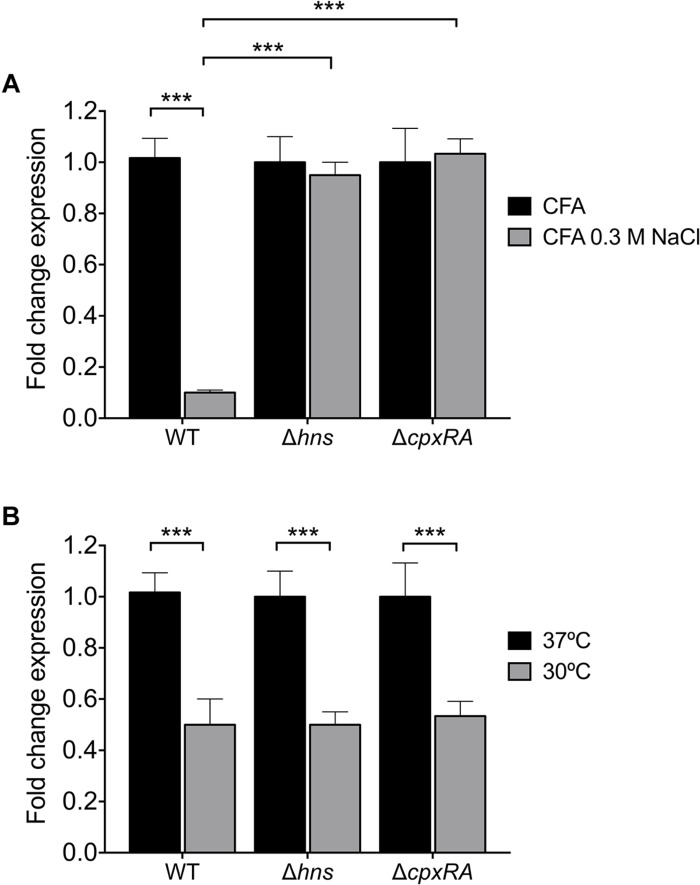
Osmo- and thermoregulation of CS3. **(A)** Transcriptional expression (RT-qPCR) of the *cstH* gene in the wild-type, Δ*hns* and Δ*cpxRA* mutants at 6 h in CFA broth supplemented with 0.3M NaCl. **(B)** Fold-change expression (RT-qPCR) of the *cstH* gene in the Δ*hns* and Δ*cpxRA* mutants as compared to E9034A. Results shown represent mean and standard deviations of three independent experiments performed. Statistically significant. ^∗∗∗^*p* < 0.001.

It was previously reported that expression of ETEC virulence factors such as the LT toxin and Longus pilus are under the influence of temperature and salt (Haycocks et al., 2015; De La Cruz et al., 2017). Since NaCl reduces CS3 gene expression (Figure 3C), we analyzed *cstH* expression in the wild-type, *hns*, and *cpxRA* backgrounds upon the addition of 0.3M NaCl to CFA broth at early stationary phase (OD_600 nm_ = 1.2) at 37°C. We found that in the absence of H-NS or CpxRA in the respective mutants, *cstH* expression was not diminished in high osmolarity medium (Figure 5A), indicating that both H-NS and CpxRA are involved in the salt-mediated repression of CS3 transcription observed in the wild-type strain.

H-NS is a thermo-regulator of virulence factors in several pathogenic bacteria (Falconi et al., 1998; Umanski et al., 2002; Ono et al., 2005; Duong et al., 2007; Ares et al., 2016; De La Cruz et al., 2017). We analyzed the regulatory effect of H-NS and CpxRA on *cstH* expression at 30 and 37°C in CFA broth at early stationary phase (OD_600 nm_ = 1.2) (Figure 5B). We found the same levels of *cstH* expression at both temperatures in the mutants and the wild-type strain, indicating that neither H-NS nor CpxRA act as thermo-regulators of *cstH* transcription. However, it is known that CS3 is favorably produced at 37°C both *in vitro* and *in vivo* suggesting the existence of other thermo-regulators that activate CS3 production in the small bowel.

### CS3 Is Required for Biofilm Formation and Adherence to Epithelial Cells

**FIGURE 6:**
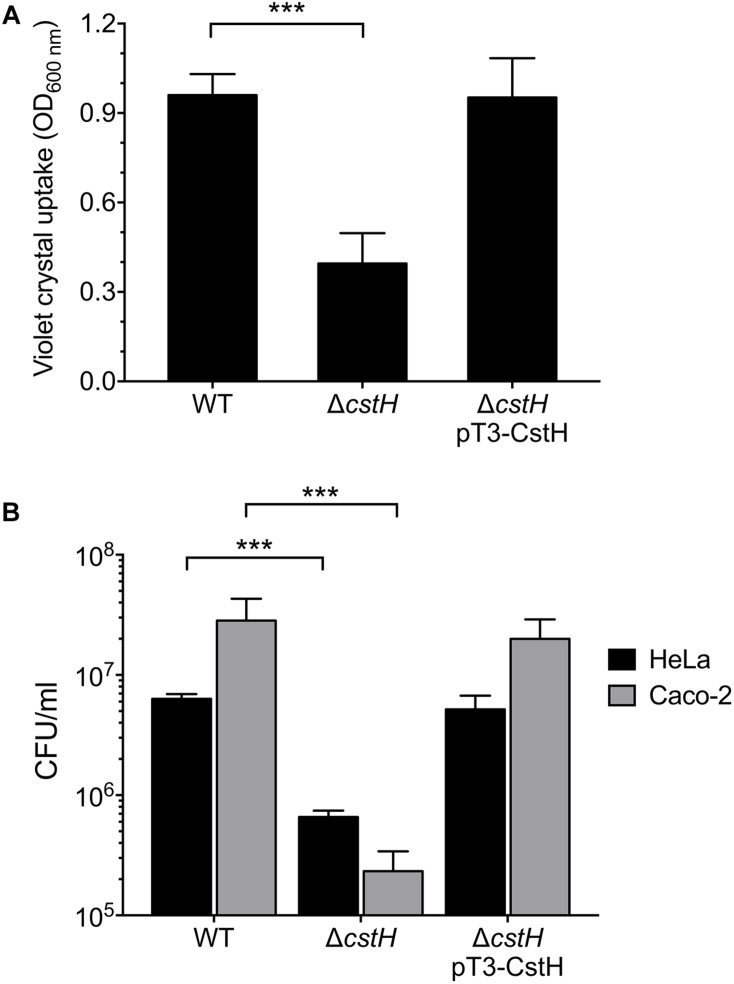
Role of the CS3 in the biofilm formation and adherence to epithelial cells. Quantification of biofilm formation **(A)** and adherent bacteria **(B)** in the wild-type (E9034A), Δ*cstH* and complemented Δ*cstH*. Quantification of biofilm production by ETEC strains growing in CFA broth as determined by adsorption of Crystal Violet at OD_595_. The indicated strains were incubated with HeLa or Caco-2 cells for 2 h and the adherent bacteria expressed as CFUs after plating serial dilutions. The experiments were performed in quadruplicate and repeated three times on different days. ^∗∗∗^*p* < 0.001.

We investigated and compared the ability of ETEC wild-type, Δ*cstH* mutant, and Δ*cstH* pT3-CstH to form biofilms. The Δ*cstH* mutant was significantly affected in biofilm formation as compared to the wild-type strain and the complemented Δ*cstH* mutant (Figure 6A). These data indicate that CS3 exerts a positive role in ETEC’s biofilm formation. Although CS3 was identified in the late 1970s, the molecular Koch’s postulates have not been fulfilled to confirm the role of CS3 in cell adherence. For example, the construction of a CS3 knock-out mutant unable to produce CS3 and to adhere to cultured epithelial cells has not been documented. Thus, we evaluated the role of CS3 in E9034’s adherence to HeLa and Caco-2 cells, testing the wild-type, its derivative isogenic *cstH* mutant, and complemented *cstH* (pT3-CstH) strains in CFA broth at early stationary phase (OD_600 nm_ = 1.2) at 37°C. Adherence of the *cstH* mutant was 10- and 121-fold reduced as compared to the wild-type strain in HeLa and Caco-2 cells, respectively (Figure 6B). The levels of adherence of the *cstH* complemented mutant were restored to wild-type levels in both HeLa and Caco-2 cells. These data confirm that CS3 is needed for adherence of ETEC to epithelial cells, particularly colonic Caco-2 cells.

## Discussion

**FIGURE 7:**
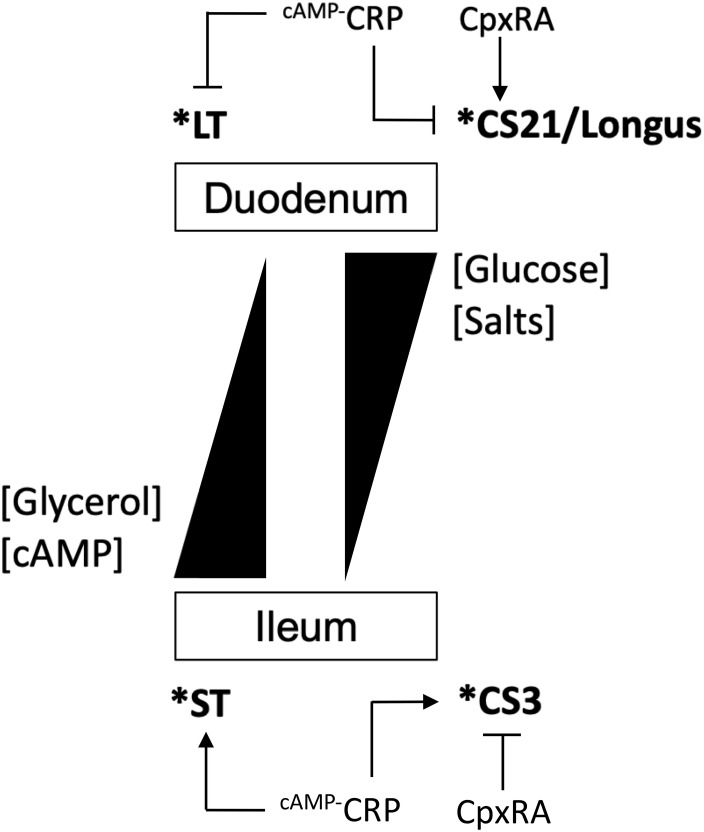
Model for the expression of CS3. Schematic of the regulation of CS3 and its differential expression with Longus (CS21) and ST and LT. High concentrations of glucose and salt activate and repress Longus/LT and CS3/ST, respectively. Salts and glucose concentrations are normally higher in the duodenum than in the ileum, while that both glycerol and cAMP are present in a contrary fashion. CpxRA and cAMP-CRP proteins differentially regulate both CS3 and Longus. H-NS represses the expression of all virulence factors and it is represented by an asterisk.

Enterotoxigenic *Escherichia coli* strains produce a repertoire of canonical CFs that are believed to promote bacterial attachment to the enterocytes in the small bowel. Typically, most CFs are encoded on large virulence plasmids along with LT and/or ST toxin genes (Qadri et al., 2000; Vidal et al., 2009; Gonzales et al., 2013; Nada et al., 2013; Torres et al., 2015; Kharat et al., 2017). Like many other bacterial virulence factors the production of ETEC CFs is affected by host and environmental stimuli such as temperature, pH, and nutritional components present in specific anatomical sites of infection. It is well-known that most CFs, except for Longus, are produced upon growth of E9034A on CFA medium at 37°C and they are less expressed in the commonly used LB broth or temperatures below 37°C. We recently reported that the production of Longus in E9034A is enhanced by the presence of calcium and addition of glucose and sodium chloride to the growth medium (De La Cruz et al., 2017). In the present study we analyzed the effect of these environmental stimuli on the expression of CS3 genes. The experimental data showed that growth of E9034A at 37°C enhanced the expression of *cstH*, but diminished at 30 and 42°C. Divalent cations-regulation of virulence factors in *Yersinia, Vibrio*, and pathogenic *E. coli* has been reported (Deng et al., 2005; Falker et al., 2006; Bilecen and Yildiz, 2009; De La Cruz et al., 2017). In the case of E9034A, we found that the presence of divalent cations such as calcium and magnesium was necessary for optimal expression of CS3 genes. In terms of effect of the carbon source in transcription, glycerol and glucose activated and repressed *cstH*, respectively. Glycerol is an antagonist molecule of glucose that positively controls the synthesis of cAMP, which is required for the transcriptional activity of CRP regulatory protein (Fic et al., 2009). Glucose is abundant in the duodenum and it is absorbed by the small intestine resulting in low concentrations of this sugar in the ileum, regulating the expression of the ST and LT enterotoxins. In contrast to glucose, glycerol is apparently accumulated toward the colon and its origin is attributed to *in situ* microbial synthesis, desquamation of epithelial cells, and low absorption of this molecule (Yuasa et al., 2003; Fujimoto et al., 2006; Ohta et al., 2006; De Weirdt et al., 2010). A model for the differential regulation of both enterotoxins was proposed in which LT is repressed while ST is enhanced in ileum, whereas the contrary occurs in the duodenum (Bodero and Munson, 2009). Judging from our data, CS3 would probably be expressed in the ileum along with ST since both virulence factors are repressed in the presence of glucose and therefore controlled by the CRP regulatory protein (Figure 7). Whereas the expression of Longus genes is increased in the presence of glucose and repressed with glycerol, the transcription control of *cstH* occurred in the opposite way.

The presence of sodium chloride repressed transcription of CS3 genes. This effect is contrary to the effect on the expression of Longus and LT genes (Haycocks et al., 2015; De La Cruz et al., 2017), Salt can control the conformation of the silencing global regulator H-NS (Dorman, 2007). H-NS represses transcription of LT, ST, and Longus genes (Yang et al., 2005; Haycocks et al., 2015; De La Cruz et al., 2017). In agreement, our data showed that H-NS also silenced *cstH* transcription. Fimbrial genes such as *csgD*, *fimA*, *papB*, *daaA* and *fanA*, are repressed in presence of salt and this effect is H-NS and CpxR-dependent (White-Ziegler et al., 2000; Schwan et al., 2002; Jubelin et al., 2005). In our study, H-NS and CpxRA regulators were required for salt-mediated CS3 repression, supporting the notion that both systems, control and integrate environmental cues such as osmolarity, in order to repress the expression of virulence genes.

Regarding CpxRA, this two-component system has been implicated in the regulation of virulence factors of some Gram-negative bacterial pathogens (Altman and Segal, 2008; Macritchie et al., 2008; Vogt et al., 2010; De La Cruz et al., 2015, 2016, 2017; Matter et al., 2018). In this study, CpxRA was found to be a repressor of CS3 expression. CRP and CpxRA seem to act antagonistically on the expression of both CS3 and Longus genes (De La Cruz et al., 2017). While CRP and CpxRA activated and repressed *cstH* expression, respectively, these regulatory proteins showed an opposite effect on *lng* genes, suggesting that the expression of CS3 and Longus genes is mutually exclusive (Figure 7). The data generated in this study are the foundation to further characterize in detail the molecular mechanisms governed by the H-NS, CpxRA, and CRP regulatory proteins that control the expression of CS3. We are currently evaluating the direct or indirect effect of these regulatory proteins on CS3 gene transcription.

Our experimental data suggest a spatio-temporal expression of CS3 and other ETEC virulence factors such as Longus and both enterotoxins in the small intestine. When ETEC reaches the duodenum, Longus and LT would be expressed in this niche. In the ileum, ETEC would switch the expression of both CS3 and ST genes. In addition to the canonical ETEC virulence factors, other surface components such as the type 1 pilus, EatA, YghJ, EaeH, EtpA have been shown to be associated with intestinal colonization in mice (Patel et al., 2004; Roy et al., 2008; Luo et al., 2014; Sheikh et al., 2014, 2017). Further studies are needed to elucidate how the expression of ETEC canonical and non-canonical virulence factors is orchestrated and synchronized during human gut colonization.

## Data Availability

All datasets generated for this study are included in the manuscript and/or the supplementary files.

## Author Contributions

MD conceived and designed the experiments. MA, JA-G, DR-V, LP, CJ-G, MJ-Q, and MC performed the experiments. MD and MA analyzed the data. MA, MA-C, JT, JG, and MD wrote the manuscript.

## Conflict of Interest Statement

The authors declare that the research was conducted in the absence of any commercial or financial relationships that could be construed as a potential conflict of interest.

## References

[B1] Altman E. Segal G. (2008). The response regulator CpxR directly regulates expression of several *Legionella pneumophila icm/dot* components as well as new translocated substrates. *J. Bacteriol.* 190 1985–1996. 10.1128/JB.01493-07 18192394 PMC2258895

[B2] Ares M. A. Fernandez-Vazquez J. L. Rosales-Reyes R. Jarillo-Quijada M. D. Von Bargen K. Torres J. (2016). H-NS nucleoid protein controls virulence features of *Klebsiella pneumoniae* by regulating the expression of Type 3 Pili and the capsule polysaccharide. *Front. Cell Infect Microbiol* 6:13. 10.3389/fcimb.2016.00013 26904512 PMC4746245

[B3] Begum Y. A. Baby N. I. Faruque A. S. Jahan N. Cravioto A. Svennerholm A. M. (2014). Shift in phenotypic characteristics of enterotoxigenic *Escherichia coli* (ETEC) isolated from diarrheal patients in Bangladesh. *PLoS Negl. Trop. Dis.* 8:e3031. 10.1371/journal.pntd.0003031 25032802 PMC4102457

[B4] Bilecen K. Yildiz F. H. (2009). Identification of a calcium-controlled negative regulatory system affecting *Vibrio cholerae* biofilm formation. *Environ. Microbiol.* 11 2015–2029. 10.1111/j.1462-2920.2009.01923.x 19397680 PMC2756528

[B5] Bodero M. D. Munson G. P. (2009). Cyclic AMP receptor protein-dependent repression of heat-labile enterotoxin. *Infect. Immun.* 77 791–798. 10.1128/IAI.00928-08 19075028 PMC2632052

[B6] Boylan M. Coleman D. C. Smyth C. J. (1987). Molecular cloning and characterization of the genetic determinant encoding CS3 fimbriae of enterotoxigenic *Escherichia coli*. *Microb. Pathog.* 2 195–209. 10.1016/0882-4010(87)90021-0 2907083

[B7] Datsenko K. A. Wanner B. L. (2000). One-step inactivation of chromosomal genes in *Escherichia coli* K-12 using PCR products. *Proc. Natl. Acad. Sci. U.S.A.* 97 6640–6645. 10.1073/pnas.120163297 10829079 PMC18686

[B8] De La Cruz M. A. Morgan J. K. Ares M. A. Yanez-Santos J. A. Riordan J. T. Giron J. A. (2016). The two-component system CpxRA negatively regulates the locus of enterocyte effacement of enterohemorrhagic *Escherichia coli* involving sigma(32) and lon protease. *Front. Cell Infect. Microbiol.* 6:11. 10.3389/fcimb.2016.00011 26904510 PMC4742615

[B9] De La Cruz M. A. Perez-Morales D. Palacios I. J. Fernandez-Mora M. Calva E. Bustamante V. H. (2015). The two-component system CpxR/A represses the expression of *Salmonella* virulence genes by affecting the stability of the transcriptional regulator HilD. *Front. Microbiol.* 6:807. 10.3389/fmicb.2015.00807 26300871 PMC4526804

[B10] De La Cruz M. A. Ruiz-Tagle A. Ares M. A. Pacheco S. Yanez J. A. Cedillo L. (2017). The expression of Longus type 4 pilus of enterotoxigenic *Escherichia coli* is regulated by LngR and LngS and by H-NS, CpxR and CRP global regulators. *Environ. Microbiol.* 19 1761–1775. 10.1111/1462-2920.13644 27943535

[B11] De Weirdt R. Possemiers S. Vermeulen G. Moerdijk-Poortvliet T. C. Boschker H. T. Verstraete W. (2010). Human faecal microbiota display variable patterns of glycerol metabolism. *FEMS Microbiol. Ecol.* 74 601–611. 10.1111/j.1574-6941.2010.00974.x 20946352

[B12] Deng W. Li Y. Hardwidge P. R. Frey E. A. Pfuetzner R. A. Lee S. (2005). Regulation of type III secretion hierarchy of translocators and effectors in attaching and effacing bacterial pathogens. *Infect. Immun.* 73 2135–2146. 10.1128/iai.73.4.2135-2146.2005 15784556 PMC1087438

[B13] Dorman C. J. (2007). H-NS, the genome sentinel. *Nat. Rev. Microbiol.* 5 157–161. 10.1038/nrmicro1598 17191074

[B14] Duong N. Osborne S. Bustamante V. H. Tomljenovic A. M. Puente J. L. Coombes B. K. (2007). Thermosensing coordinates a cis-regulatory module for transcriptional activation of the intracellular virulence system in *Salmonella enterica* serovar Typhimurium. *J. Biol. Chem.* 282 34077–34084. 10.1074/jbc.m707352200 17895240

[B15] Evans D. G. Evans D. J. Jr. Tjoa W. (1977). Hemagglutination of human group A erythrocytes by enterotoxigenic *Escherichia coli* isolated from adults with diarrhea: correlation with colonization factor. *Infect. Immun.* 18 330–337. 336541 10.1128/iai.18.2.330-337.1977 PMC421235

[B16] Falconi M. Colonna B. Prosseda G. Micheli G. Gualerzi C. O. (1998). Thermoregulation of *Shigella* and *Escherichia coli* EIEC pathogenicity. A temperature-dependent structural transition of DNA modulates accessibility of virF promoter to transcriptional repressor H-NS. *EMBO J.* 17 7033–7043. 10.1093/emboj/17.23.7033 9843508 PMC1171051

[B17] Falker S. Schmidt M. A. Heusipp G. (2006). Altered Ca(2+) regulation of Yop secretion in *Yersinia enterocolitica* after DNA adenine methyltransferase overproduction is mediated by Clp-dependent degradation of LcrG. *J. Bacteriol.* 188 7072–7081. 10.1128/jb.00583-06 17015646 PMC1636222

[B18] Fic E. Bonarek P. Gorecki A. Kedracka-Krok S. Mikolajczak J. Polit A. (2009). cAMP receptor protein from *Escherichia coli* as a model of signal transduction in proteins–a review. *J. Mol. Microbiol. Biotechnol.* 17 1–11. 10.1159/000178014 19033675

[B19] Fleckenstein J. M. Hardwidge P. R. Munson G. P. Rasko D. A. Sommerfelt H. Steinsland H. (2010). Molecular mechanisms of enterotoxigenic *Escherichia coli* infection. *Microbes Infect.* 12 89–98. 10.1016/j.micinf.2009.10.002 19883790 PMC10647112

[B20] Fujimoto N. Inoue K. Hayashi Y. Yuasa H. (2006). Glycerol uptake in HCT-15 human colon cancer cell line by Na(+)-dependent carrier-mediated transport. *Biol. Pharm. Bull.* 29 150–154. 10.1248/bpb.29.150 16394529

[B21] Giron J. A. Levine M. M. Kaper J. B. (1994). Longus: a long pilus ultrastructure produced by human enterotoxigenic *Escherichia coli*. *Mol. Microbiol.* 12 71–82. 10.1111/j.1365-2958.1994.tb00996.x 7914665

[B22] Giron J. A. Xu J. G. Gonzalez C. R. Hone D. Kaper J. B. Levine M. M. (1995). Simultaneous expression of CFA/I and CS3 colonization factor antigens of enterotoxigenic *Escherichia coli* by delta *aroC*, delta *aroD Salmonella typhi* vaccine strain CVD 908. *Vaccine* 13 939–946. 10.1016/0264-410x(95)00003-j 7483768

[B23] Gonzales L. Sanchez S. Zambrana S. Iniguez V. Wiklund G. Svennerholm A. M. (2013). Molecular characterization of enterotoxigenic *Escherichia coli* isolates recovered from children with diarrhea during a 4-year period (2007 to 2010) in Bolivia. *J. Clin. Microbiol.* 51 1219–1225. 10.1128/JCM.02971-12 23390275 PMC3666779

[B24] Guo M. Wang H. Xie N. Xie Z. (2015). Positive effect of carbon sources on natural transformation in *Escherichia coli*: role of low-level cyclic AMP (cAMP)-cAMP receptor protein in the derepression of *rpoS*. *J. Bacteriol.* 197 3317–3328. 10.1128/JB.00291-15 26260461 PMC4573726

[B25] Hall R. H. Maneval D. R. Jr. Collins J. H. Theibert J. L. Levine M. M. (1989). Purification and analysis of colonization factor antigen I, coli surface antigen 1, and coli surface antigen 3 fimbriae from enterotoxigenic *Escherichia coli*. *J. Bacteriol.* 171 6372–6374. 10.1128/jb.171.11.6372-6374.1989 2572583 PMC210515

[B26] Haycocks J. R. Sharma P. Stringer A. M. Wade J. T. Grainger D. C. (2015). The molecular basis for control of ETEC enterotoxin expression in response to environment and host. *PLoS Pathog.* 11:e1004605. 10.1371/journal.ppat.1004605 25569153 PMC4287617

[B27] Jahn C. E. Charkowski A. O. Willis D. K. (2008). Evaluation of isolation methods and RNA integrity for bacterial RNA quantitation. *J. Microbiol. Methods* 75 318–324. 10.1016/j.mimet.2008.07.004 18674572

[B28] Jalajakumari M. B. Thomas C. J. Halter R. Manning P. A. (1989). Genes for biosynthesis and assembly of CS3 pili of CFA/II enterotoxigenic *Escherichia coli*: novel regulation of pilus production by bypassing an amber codon. *Mol. Microbiol.* 3 1685–1695. 10.1111/j.1365-2958.1989.tb00154.x 2576094

[B29] Jubelin G. Vianney A. Beloin C. Ghigo J. M. Lazzaroni J. C. Lejeune P. (2005). CpxR/OmpR interplay regulates curli gene expression in response to osmolarity in *Escherichia coli*. *J. Bacteriol.* 187 2038–2049. 10.1128/jb.187.6.2038-2049.2005 15743952 PMC1064031

[B30] Kansal R. Rasko D. A. Sahl J. W. Munson G. P. Roy K. Luo Q. (2013). Transcriptional modulation of enterotoxigenic *Escherichia coli* virulence genes in response to epithelial cell interactions. *Infect. Immun.* 81 259–270. 10.1128/IAI.00919-12 23115039 PMC3536156

[B31] Khalil I. A. Troeger C. Blacker B. F. Rao P. C. Brown A. Atherly D. E. (2018). Morbidity and mortality due to *Shigella* and enterotoxigenic *Escherichia coli* diarrhoea: the global burden of disease study 1990-2016. *Lancet Infect. Dis.* 18 1229–1240. 10.1016/S1473-3099(18)30475-4 30266330 PMC6202441

[B32] Kharat V. B. Ahmed M. Jiang Z. D. Riddle M. S. Dupont H. L. (2017). Colonization factors in enterotoxigenic *Escherichia coli* strains in travelers to Mexico, Guatemala, and India compared with children in houston, Texas. *Am. J. Trop. Med. Hyg.* 96 83–87. 10.4269/ajtmh.16-0405 28077742 PMC5239714

[B33] Knutton S. Lloyd D. R. Candy D. C. Mcneish A. S. (1985). Adhesion of enterotoxigenic *Escherichia coli* to human small intestinal enterocytes. *Infect. Immun.* 48 824–831. 2860070 10.1128/iai.48.3.824-831.1985 PMC261277

[B34] Kolb A. Busby S. Buc H. Garges S. Adhya S. (1993). Transcriptional regulation by cAMP and its receptor protein. *Annu. Rev. Biochem.* 62 749–795. 10.1146/annurev.biochem.62.1.749 8394684

[B35] Levine M. M. (1987). *Escherichia coli* that cause diarrhea: enterotoxigenic, enteropathogenic, enteroinvasive, enterohemorrhagic, and enteroadherent. *J. Infect. Dis.* 155 377–389. 10.1093/infdis/155.3.377 3543152

[B36] Levine M. M. Ristaino P. Marley G. Smyth C. Knutton S. Boedeker E. (1984). Coli surface antigens 1 and 3 of colonization factor antigen II-positive enterotoxigenic *Escherichia coli*: morphology, purification, and immune responses in humans. *Infect. Immun.* 44 409–420. 6370866 10.1128/iai.44.2.409-420.1984 PMC263534

[B37] Livak K. J. Schmittgen T. D. (2001). Analysis of relative gene expression data using real-time quantitative PCR and the 2(-Delta Delta C(T)) Method. *Methods* 25 402–408. 10.1006/meth.2001.1262 11846609

[B38] Luo Q. Kumar P. Vickers T. J. Sheikh A. Lewis W. G. Rasko D. A. (2014). Enterotoxigenic *Escherichia coli* secretes a highly conserved mucin-degrading metalloprotease to effectively engage intestinal epithelial cells. *Infect. Immun.* 82 509–521. 10.1128/IAI.01106-13 24478067 PMC3911403

[B39] Macritchie D. M. Ward J. D. Nevesinjac A. Z. Raivio T. L. (2008). Activation of the Cpx envelope stress response down-regulates expression of several locus of enterocyte effacement-encoded genes in enteropathogenic *Escherichia coli*. *Infect. Immun.* 76 1465–1475. 10.1128/IAI.01265-07 18227171 PMC2292881

[B40] Madhavan T. P. Sakellaris H. (2015). Colonization factors of enterotoxigenic *Escherichia coli*. *Adv. Appl. Microbiol.* 90 155–197. 10.1016/bs.aambs.2014.09.003 25596032

[B41] Manning P. A. Timmis K. N. Stevenson G. (1985). Colonization factor antigen II (CFA/II) of enterotoxigenic *Escherichia coli*: molecular cloning of the CS3 determinant. *Mol. Gen. Genet.* 200 322–327. 10.1007/bf00425443 2863737

[B42] Matter L. B. Ares M. A. Abundes-Gallegos J. Cedillo M. L. Yanez J. A. Martinez-Laguna Y. (2018). The CpxRA stress response system regulates virulence features of avian pathogenic *Escherichia coli*. *Environ. Microbiol.* 20 3363–3377. 10.1111/1462-2920.14368 30062827

[B43] Mayer M. P. (1995). A new set of useful cloning and expression vectors derived from pBlueScript. *Gene* 163 41–46. 10.1016/0378-1119(95)00389-n 7557476

[B44] Mellies J. L. Barron A. M. (2006). Virulence gene regulation in *Escherichia coli*. *EcoSal Plus* 2 1–36. 10.1128/ecosalplus.8.9.1 26443571

[B45] Mirhoseini A. Amani J. Nazarian S. (2018). Review on pathogenicity mechanism of enterotoxigenic *Escherichia coli* and vaccines against it. *Microb. Pathog.* 117 162–169. 10.1016/j.micpath.2018.02.032 29474827

[B46] Munson G. P. (2013). Virulence regulons of enterotoxigenic *Escherichia coli*. *Immunol. Res.* 57 229–236. 10.1007/s12026-013-8453-4 24203442

[B47] Nada R. A. Armstrong A. Shaheen H. I. Nakhla I. Sanders J. W. Riddle M. S. (2013). Phenotypic and genotypic characterization of enterotoxigenic *Escherichia coli* isolated from U.S. Military personnel participating in Operation Bright Star, Egypt, from 2005 to 2009. *Diagn. Microbiol. Infect. Dis.* 76 272–277. 10.1016/j.diagmicrobio.2013.03.028 23639795

[B48] Ohta K. Y. Inoue K. Hayashi Y. Yuasa H. (2006). Carrier-mediated transport of glycerol in the perfused rat small intestine. *Biol. Pharm. Bull.* 29 785–789. 10.1248/bpb.29.785 16595918

[B49] Ono S. Goldberg M. D. Olsson T. Esposito D. Hinton J. C. Ladbury J. E. (2005). H-NS is a part of a thermally controlled mechanism for bacterial gene regulation. *Biochem. J.* 391 203–213. 10.1042/bj20050453 15966862 PMC1276917

[B50] Patel S. K. Dotson J. Allen K. P. Fleckenstein J. M. (2004). Identification and molecular characterization of EatA, an autotransporter protein of enterotoxigenic *Escherichia coli*. *Infect. Immun.* 72 1786–1794. 10.1128/iai.72.3.1786-1794.2004 14977988 PMC356008

[B51] Qadri F. Das S. K. Faruque A. S. Fuchs G. J. Albert M. J. Sack R. B. (2000). Prevalence of toxin types and colonization factors in enterotoxigenic *Escherichia coli* isolated during a 2-year period from diarrheal patients in Bangladesh. *J. Clin. Microbiol.* 38 27–31. 10618058 10.1128/jcm.38.1.27-31.2000 PMC86010

[B52] Qadri F. Svennerholm A. M. Faruque A. S. Sack R. B. (2005). Enterotoxigenic *Escherichia coli* in developing countries: epidemiology, microbiology, clinical features, treatment, and prevention. *Clin. Microbiol. Rev.* 18 465–483. 10.1128/cmr.18.3.465-483.2005 16020685 PMC1195967

[B53] Rothenbacher F. P. Zhu J. (2014). Efficient responses to host and bacterial signals during *Vibrio cholerae* colonization. *Gut Microbes* 5 120–128. 10.4161/gmic.26944 24256715 PMC4049929

[B54] Roy K. Hamilton D. Allen K. P. Randolph M. P. Fleckenstein J. M. (2008). The EtpA exoprotein of enterotoxigenic *Escherichia coli* promotes intestinal colonization and is a protective antigen in an experimental model of murine infection. *Infect. Immun.* 76 2106–2112. 10.1128/IAI.01304-07 18285493 PMC2346670

[B55] Schwan W. R. Lee J. L. Lenard F. A. Matthews B. T. Beck M. T. (2002). Osmolarity and pH growth conditions regulate fim gene transcription and type 1 pilus expression in uropathogenic *Escherichia coli*. *Infect. Immun.* 70 1391–1402. 10.1128/iai.70.3.1391-1402.2002 11854225 PMC127777

[B56] Sheikh A. Luo Q. Roy K. Shabaan S. Kumar P. Qadri F. (2014). Contribution of the highly conserved EaeH surface protein to enterotoxigenic *Escherichia coli* pathogenesis. *Infect. Immun.* 82 3657–3666. 10.1128/IAI.01890-14 24935979 PMC4187836

[B57] Sheikh A. Rashu R. Begum Y. A. Kuhlman F. M. Ciorba M. A. Hultgren S. J. (2017). Highly conserved type 1 pili promote enterotoxigenic *E. coli pathogen-host interactions*. *PLoS Negl. Trop. Dis.* 11:e0005586. 10.1371/journal.pntd.0005586 28531220 PMC5456409

[B58] Torres O. R. Gonzalez W. Lemus O. Pratdesaba R. A. Matute J. A. Wiklund G. (2015). Toxins and virulence factors of enterotoxigenic *Escherichia coli* associated with strains isolated from indigenous children and international visitors to a rural community in Guatemala. *Epidemiol. Infect.* 143 1662–1671. 10.1017/S0950268814002295 25233938 PMC4416357

[B59] Umanski T. Rosenshine I. Friedberg D. (2002). Thermoregulated expression of virulence genes in enteropathogenic *Escherichia coli*. *Microbiology* 148 2735–2744. 10.1099/00221287-148-9-2735 12213920

[B60] Vidal R. M. Muhsen K. Tennant S. M. Svennerholm A. M. Sow S. O. Sur D. (2019). Colonization factors among enterotoxigenic *Escherichia coli* isolates from children with moderate-to-severe diarrhea and from matched controls in the Global Enteric Multicenter Study (GEMS). *PLoS Negl. Trop. Dis.* 13:e0007037. 10.1371/journal.pntd.0007037 30608930 PMC6343939

[B61] Vidal R. M. Valenzuela P. Baker K. Lagos R. Esparza M. Livio S. (2009). Characterization of the most prevalent colonization factor antigens present in Chilean clinical enterotoxigenic *Escherichia coli* strains using a new multiplex polymerase chain reaction. *Diagn. Microbiol. Infect. Dis.* 65 217–223. 10.1016/j.diagmicrobio.2009.07.005 19733027

[B62] Vogt S. L. Nevesinjac A. Z. Humphries R. M. Donnenberg M. S. Armstrong G. D. Raivio T. L. (2010). The Cpx envelope stress response both facilitates and inhibits elaboration of the enteropathogenic *Escherichia coli* bundle-forming pilus. *Mol. Microbiol.* 76 1095–1110. 10.1111/j.1365-2958.2010.07145.x 20444097 PMC2904494

[B63] Vogt S. L. Raivio T. L. (2012). Just scratching the surface: an expanding view of the Cpx envelope stress response. *FEMS Microbiol. Lett.* 326 2–11. 10.1111/j.1574-6968.2011.02406.x 22092948

[B64] White-Ziegler C. A. Villapakkam A. Ronaszeki K. Young S. (2000). H-NS controls pap and daa fimbrial transcription in *Escherichia coli* in response to multiple environmental cues. *J. Bacteriol.* 182 6391–6400. 10.1128/jb.182.22.6391-6400.2000 11053383 PMC94785

[B65] Yakhchali B. Manning P. A. (1997). Epitope analysis of the CS3 fimbrial subunit of human enterotoxigenic *Escherichia coli* and the construction of novel CS3::ST and CS3::LT-B immunogens. *Behring Inst. Mitt.* 98 124–134. 9382733

[B66] Yang J. Tauschek M. Strugnell R. Robins-Browne R. M. (2005). The H-NS protein represses transcription of the *eltAB* operon, which encodes heat-labile enterotoxin in enterotoxigenic *Escherichia coli*, by binding to regions downstream of the promoter. *Microbiology* 151 1199–1208. 10.1099/mic.0.27734-0 15817787

[B67] Yuasa H. Hamamoto K. Dogu S. Y. Marutani T. Nakajima A. Kato T. (2003). Saturable absorption of glycerol in the rat intestine. *Biol. Pharm. Bull.* 26 1633–1636. 10.1248/bpb.26.1633 14600418

